# Multimodal MRI reveals three-tiered pathological co-alterations in prolonged disorders of consciousness: structural disconnection, network disintegration, and regional hyperconnectivity

**DOI:** 10.3389/fneur.2026.1797576

**Published:** 2026-06-09

**Authors:** Xiyong Wang, Yi Yin, Xianbin Wang, Shuang Wu

**Affiliations:** 1 Department of Rehabilitation Medicine, Affiliated Hospital of Guizhou Medical University, Guiyang, China; 2 Department of Radiology, Affiliated Hospital of Guizhou Medical University, Guiyang, China

**Keywords:** fractional anisotropy, functional connectivity, multimodal magnetic resonance imaging, neuroimaging biomarkers, prolonged disorders of consciousness

## Abstract

**Background:**

Clinical diagnosis of prolonged disorders of consciousness (pDoC) relies primarily on behavioral scales, which lack sensitivity to covert cerebral cognitive activity and carry a risk of misdiagnosis. Few studies integrate structural and functional brain abnormalities to explore pDoC. The objective of this study was to employ resting-state functional magnetic resonance imaging (rs-fMRI) with diffusion tensor imaging (DTI) to delineate multimodal neuroimaging abnormalities in pDoC, clarify its neural mechanisms, and identify potential neuroimaging biomarkers.

**Methods:**

Eighteen patients with pDoC and 20 healthy controls (HCs) underwent rs-fMRI and DTI acquisition. Key metrics included fractional anisotropy (FA), amplitude of low-frequency fluctuations (ALFF), fractional ALFF (fALFF), regional homogeneity (ReHo), and functional connectivity (FC). Correlations between these metrics and Coma Recovery Scale-Revised (CRS-R) scores were analyzed.

**Results:**

Compared with HCs, pDoC patients exhibited reduced FA in the left anterior corona radiata (ACR-L) (*p* < 0.05), altered ALFF/fALFF/ReHo in prefrontal, cerebellar, and limbic regions, and disrupted FC within higher-order cortical networks. CRS-R scores positively correlated with ACR-L FA and prefrontal ReHo, and negatively correlated with cerebellar and limbic hyperactivity (*p* < 0.05).

**Conclusion:**

Patients with pDoC demonstrate three simultaneous tiers of pathological co-alterations: extensive white matter structural disruption with peak significance in ACR-L; disintegration of higher-order cortical networks; patterns of hyperactivity and hyperconnectivity in the cerebellum, limbic system, and angular gyrus. Our findings provide a framework for understanding the pathophysiological mechanisms underlying consciousness impairment in pDoC. The neuroimaging biomarkers found in this study facilitate objective pDoC assessment and prognostic evaluation.

## 1 Introduction

Prolonged disorders of consciousness (pDoC) refer to a clinical syndrome that emerges following severe acquired brain injury, marked by impaired wakefulness, awareness perception, and environmental interaction lasting over 28 days (1). It mainly includes Unresponsive Wakefulness Syndrome (UWS) and Minimally Conscious State (MCS) (2). UWS patients have sleep–wake cycles and may open their eyes spontaneously, but lack behavioral signs of self or environmental awareness. They only show reflexive movements, with no purposeful responses mediated by the cerebral cortex. In contrast, MCS patients exhibit minimal, fluctuating behavioral awareness—such as visual tracking, pain localization, command-following motor responses, and simple communication signals. Prognostically, MCS patients have a better recovery outlook than UWS patients: around half of post-traumatic MCS patients regain consciousness within 1 year, while fewer than 10% of long-term UWS patients achieve recovery (2, 3). Accurate evaluation and effective intervention for consciousness recovery pose significant challenges in clinical neuroscience (4). Despite the widespread adoption of the Coma Recovery Scale-Revised (CRS-R) for behavioral assessment (3), its applicability is hindered by motor deficits, sensory impairments, and inter-assessor variability. These limitations often lead to misdiagnosis of many patients with hidden cognitive abilities. Such issues with diagnosis undermine the precision of research understanding and also impact prognosis stratification and treatment decision-making (5).

Over the past 20 years, methods using neuroimaging techniques have changed the ability to examine brain features relating to consciousness. Functional magnetic resonance imaging (fMRI), electroencephalography (EEG), and positron emission tomography (PET) technologies enable the detection of latent consciousness in patients with severe brain injuries and elucidate the neural mechanisms preserved in such cases (6–8). These approaches have provided understanding of features in conditions of consciousness and have informed assessment of outcomes and recovery patterns (9). The Resting-state fMRI (rs-fMRI) paradigm, which analyzes spontaneous BOLD signal fluctuations at rest, facilitates the exploration of intrinsic functional architecture. Important metrics derived from rs-fMRI include amplitude of low-frequency fluctuations (ALFF), which reflects the strength of local spontaneous neural activity, functional connectivity (FC), which measures temporal correlations between distant brain regions, and regional homogeneity (ReHo), which assesses the temporal synchronization of nearby neural activity. Together, these metrics provide a multi-scale perspective on brain function, ranging from local activity to large-scale network interactions (10, 11). Data from numerous studies indicate that individuals with consciousness-related conditions exhibit decreased functional integration and disrupted organization in networks associated with consciousness processing. These disruptions are most pronounced in networks essential for conscious awareness, including the dorsal attention (DAN), frontoparietal (FPN), and default mode (DMN) networks, along with the ventral attention (VAN) network (12, 13). The reduction of connections within networks, combined with patterns that differ across networks, provides a main factor in the loss of conscious processing. In addition to these findings on function, diffusion tensor imaging (DTI) examines structure in the brain by measuring the direction that water molecules move in tissue (14). This approach reveals disconnection in structure within networks that support consciousness. Decreased fractional anisotropy (FA) values provide an anatomical substrate for impaired consciousness. The structure of pathways that relay information, including pathways between regions that process information and pathways that connect different regions across the brain, often shows damage. This damage to structure reduces the effectiveness of transfer of information across distances in the brain and increases the disruption of function across networks that occur at large scale in the brain.

Studies focusing on a single modality tend to offer incomplete insights, as previous research has mainly been limited to isolated or parallel descriptions of structural and functional abnormalities, this approach fails to integrate anomalies across different scales into a cohesive pathophysiological framework. Key questions still remain unanswered: how structural and functional brain abnormalities interact as concurrent patterns associated with consciousness impairment, and how these cross-modal perturbations collectively underpin the core clinical deficits in pDoC. Addressing these gaps is essential to move beyond phenomenological descriptions toward a mechanistic understanding of pDoC pathogenesis. Although calls to translate neuroimaging findings into clinical tools are growing (15), developing a comprehensive theoretical model remains elusive. Some studies, while valuable in revealing functional disturbances in cortical and subcortical regions of people with severe traumatic brain injury, focus predominantly on functional alterations without systematically incorporating structural correlates (16). An approach that integrates different forms of data and provides explanation is still absent. The trend that involves combining different forms of data provides a solution that shows potential: combining fMRI with DTI has demonstrated potential for classifying disorders of consciousness show potential for separating different forms of disorder of consciousness (17), suggesting a path that uses methods to address gaps that currently appear. To address these gaps, this work uses a framework that integrates different forms of magnetic resonance imaging, combining the CRS-R, rs-fMRI, and DTI. This study is driven by three main goals: systematically analyzing the patterns of change in both the structure and function of individuals with prolonged disorders of consciousness; examining relationships between measures from different forms of imaging and scores from assessment of behavior using the CRS-R to establish that the measures relate to findings that appear in clinical settings; and finding measures from imaging that provide new indicators for determining the degree of impairment in consciousness, establishing a basis for predicting outcomes in clinical settings (18). Using this approach that combines different forms of data, the work aims to explain mechanisms that produce prolonged disorder of consciousness at different levels, providing information for efforts to develop interventions that target particular mechanisms in the future (19).

## 2 Materials and methods

### 2.1 Participants

This study was conducted at our institution, the Affiliated Hospital of Guizhou Medical University. From May 2024 to May 2025, we prospectively recruited 30 patients with pDoC. A control group consisting of 20 healthy volunteers matched for age and sex was recruited concurrently. During MRI acquisition, nine patients were excluded due to uncontrollable motion artifacts. A further three patients were excluded at the preprocessing stage for head motion exceeding 3 mm. Consequently, the final analytical cohort comprised 18 pDoC patients (Table 1).

**Table 1:** Demographic and clinical characteristics of patients with pDoC.

Patient	Sex	Age/y	Etiology	Duration/m	CRS-R
MCS1	Male	58	Hemorrhage	3	11
MCS2	Female	18	TBI	2	11
MCS3	Male	78	TBI	6	8
MCS4	Male	71	Hemorrhage	1	8
MCS5	Female	50	Hemorrhage	1	12
MCS6	Female	46	Neoplasms	6	13
MCS7	Female	45	Hemorrhage	9	13
MCS8	Male	36	Hemorrhage	4	8
UWS1	Male	52	Hemorrhage	2	3
UWS2	Male	45	Hemorrhage	2	0
UWS3	Female	57	Hemorrhage	3	0
UWS4	Male	40	TBI	6	1
UWS5	Male	67	Hemorrhage	5	3
UWS6	Female	45	Hemorrhage	1	6
UWS7	Female	46	Neoplasms	5	2
UWS8	Male	36	Hemorrhage	1	1
UWS9	Male	60	Hemorrhage	1	1
UWS10	Male	52	Hemorrhage	12	7

Inclusion criteria for patients with pDoC included the following: (1) age ≥18 years; (2) a first-episode brain injury of either traumatic (TBI), hemorrhagic, or hypoxic–ischemic origin, confirmed by neuroimaging (CT/MRI); (3) a clinical diagnosis of pDoC according to the 2020 European Academy of Neurology consensus guidelines, with a documented consciousness impairment duration of at least 28 days; (4) hemodynamic and respiratory stability; (5) provision of written informed consent by the patient’s legally authorized representative.

Exclusion criteria were as follows: (1) consciousness impairment caused by metabolic factors, toxic factors, or neurodegenerative conditions; (2) history of neurological or psychiatric disorders; (3) presence of MRI scan contraindications; (4) unstable intracranial conditions, including ongoing active hemorrhage, progressive cerebral edema, or significantly high intracranial pressure; (5) other severe systemic illnesses that coexist, such as advanced cancer, severe liver or kidney dysfunction, or uncontrolled heart arrhythmias; (6) use of medications that affect neural activity or cerebral metabolism within 7 days before the study; (7) significant sensory or motor deficits that could seriously affect behavioral assessment. All patients undergo CRS-R assessments, which are conducted by professionals.

Healthy controls (HCs) were also excluded if they met any of the following criteria: a documented history of substance abuse; use within the past month of any medication known to affect central nervous system activity; or any chronic neurological or systemic disorder known to compromise cerebral integrity or function.

Ethical approval was granted by the Ethics Committee of Guizhou Medical University (Approval No. 2024013K). The study was also prospectively registered at the Chinese Clinical Trial Registry (Registration No. ChiCTR2400088110). All research activities involving human participants strictly adhered to the ethical standards set forth in the Declaration of Helsinki.

### 2.2 MRI data acquisition

All MRI scans were acquired using a GE Discovery MR 750 3.0T scanner (GE Healthcare, Fairfield, CT, USA) using its 32-channel head coil. During scanning, pDoC patients were not given any sedatives to prevent influencing the neural activity measurements. HCs were guided to maintain stillness with eyes gently closed, stay awake, and actively reduce head and body movement.

For high resolution brain anatomy we acquired a T1 weighted 3D fast spoiled gradient echo (FSPGR) sequence. Sequence parameters: repetition time (TR) = 8.6 ms, echo time (TE) = 3.2 ms, flip angle = 12° field of view (FOV) = 256 × 256 mm, matrix size = 256 × 256, voxels size = 1 × 1 × 1 mm^3^, total number of sagittal slices = 176. After spatial standardization, we obtained high anatomical resolution images.

The rs-fMRI data were obtained using gradient-recalled echo planar imaging (GRE-EPI). The acquisition parameters were: TR = 2,000 ms, TE = 25 ms, flip angle = 90°, FOV = 240 × 240 mm^2^, matrix = 64 × 64, slice thickness = 3.6 mm, no inter slice gap. Forty axial slices per volume were acquired. The total duration of the rs-fMRI acquisition was 7 min (210 volumes), and this was long enough to sample low frequency neural oscillations.

To assess white matter microstructure, DTI was performed with a single-shot spin-echo echo-planar imaging (SS-EPI) sequence. The scan parameters were set as follows: TR = 8,500 ms, TE = 80 ms, FOV = 240 × 240 mm^2^, matrix = 128 × 128, 60 axial slices with slice thickness = 2 mm. Diffusion-weighted images were obtained along 32 directions (b = 1,000 s/mm^2^). One non-diffusion-weighted volume (b = 0 s/mm^2^) was also acquired for distortion correction.

### 2.3 Data Preprocessing

#### 2.3.1 Structural DTI data

The diffusion-weighted images (DWIs) were processed using FSL 6.0 (20), software for diffusion tensor imaging analysis. The raw DWI data were examined for motion artifacts and signal loss using DTIPrep (21) software. Distortions caused by eddy currents and head movement were corrected using FSL’s eddy tool (22), which uses methods combining alignment and warping to reduce deviations from movement and scanner gradients. Non-diffusion-weighted (*b* = 0) volumes provided the reference for correcting distortion. Tissues outside the brain, such as the skull, scalp, and cerebrospinal fluid (CSF), were removed using bet (Brain Extraction Tool) to improve the accuracy of subsequent registration and voxel-wise analysis (23). The diffusion tensor model was then applied using dtifit, producing maps of FA, mean diffusivity (MD), axial diffusivity (AD), and radial diffusivity (RD) for each individual. FA is highly sensitive to white matter microstructural integrity, so this measure was selected for group analysis (24). The FA maps were normalized to the Montreal Neurological Institute (MNI) 152 standard space (2 × 2 × 2 mm^3^ isotropic resolution). Normalization was performed using linear affine transformation and nonlinear registration with FNIRT, which aligned images across individuals for group analysis.

#### 2.3.2 Functional rs-fMRI data

We processed rs-fMRI data using the DPABI toolbox, which runs on MATLAB R2021b (25). The workflow started with converting raw DICOM files to NIFTI format. After removing the first 10 volumes to stabilize the magnetic field, slice timing correction, then head motion correction by rigid transformation to estimate six motion parameters, and registration of functional images with individual T1-weighted structural images (26). Next, the realigned images underwent spatial normalization to the Montreal Neurological Institute (MNI) template and were then resampled to a voxel size of 3 × 3 × 3 mm^3^. To reduce non-neural signal interference, we used linear regression to remove average signals from white matter and cerebrospinal fluid, and regressed out 24 Friston motion parameters. We did not apply global signal regression to avoid spurious negative correlations (27). We then applied a temporal band-pass filter (0.01–0.1 Hz) to retain low-frequency fluctuations, and used a 4 mm full-width at half-maximum (FWHM) Gaussian kernel for spatial smoothing to improve signal-to-noise ratio. All images underwent rigid-body, affine, and nonlinear diffeomorphic spatial normalization to the standard MNI152 template. Ventricular and cerebral atrophy masks were incorporated during preprocessing to minimize anatomical distortion interference. Qualitative evaluation of individual deformation fields confirmed no extreme registration bias in the included cohort.

### 2.4 Analyses of FA, ALFF, fALFF, ReHo, and FC

We generated maps of FA, ALFF, fractional ALFF (fALFF), ReHo, and FC using DPABI V5.4. FA maps were obtained by fitting a voxel-wise diffusion tensor model to the DTI data preprocessing results (28). ALFF was calculated for each voxel as the square root of the mean power spectrum within the 0.01–0.1 Hz band, providing a measure of the intensity of spontaneous regional neuronal activity (29). fALFF was defined as the proportion of power within the low-frequency range (0.01–0.1 Hz) relative to the total power over the full frequency spectrum (0–0.25 Hz). Such normalization boosts the specificity toward neuronal activity while mitigating interference from physiological noise (30). ReHo was developed to evaluate local neural synchrony. This metric measures how similar the BOLD signal time courses are between each voxel and its 26 closest neighbors, using Kendall’s coefficient of concordance. ReHo is an indicator of how well local neural ensembles work together and shows how well adjacent resting-state networks are functioning (31). FC was assessed using a seed-based correlation approach. The seeds comprised the seven large-scale resting-state networks defined by Yeo et al. (32): the FPN, DAN, DMN, limbic network (LIM), sensorimotor network (SMN), VAN, and visual network (VN). We first extracted the mean time series for every network. Next, a whole-brain voxelwise correlation analysis was performed by computing the cross-correlation between each network’s time series and every other voxel’s time series. Finally, all correlation coefficients underwent Fisher’s r-to-z transformation to yield z-scores, normalizing the data for subsequent group-level statistical tests.

### 2.5 Statistical analysis

Statistical analysis of demographic characteristics between the pDoC group and HCs was performed using SPSS 26.0 (IBM Corporation, USA). For comparisons of continuous variables with normal distribution, independent samples t-tests were applied; for non-normally distributed data, Mann–Whitney U tests were used. Group differences in gender were evaluated with the chi-square test. A *p*-value threshold of 0.05 defined statistical significance. Voxel-wise comparisons of FA, ALFF, fALFF, ReHo, and FC between groups were performed using DPABI software with a two-sample t-test, controlling for age, gender, and years of education as covariates. Statistical significance was defined at a cluster-level threshold of *p* < 0.05, following inference with threshold-free cluster enhancement (TFCE) with 5,000 permutations (33). White matter clusters were delineated according to the JHU ICBM-DTI-81 atlas (34). For subsequent quantitative analysis, mean FA values were then derived from nine major fiber tracts within these clusters; functional metric clusters were referenced to the Automated Anatomical Labeling atlas version 3 (AAL3). Pearson correlation analysis, conducted in SPSS, was employed to evaluate correlations between the total CRS-R scores and the mean values of neuroimaging metrics that had been extracted from regions showing significant intergroup differences (two-tailed *p* < 0.05). Partial correlations were used to explore relationships between imaging metrics and CRS-R scores while controlling for diagnostic subgroup (UWS/MCS).

## 3 Results

### 3.1 Demographic and clinical characteristics

At baseline demographics, the pDoC group and HCs group showed no significant differences in age, gender, or educational attainment, as detailed in Table 2.

**Table 2:** Demographic and clinical characteristics comparison between pDoC patients and HCs.

Variable	pDoC (*n* = 18) Mean ± SD	HC (*n* = 20) Mean ± SD	*T/χ^2^*	*p* value
Age (years)	49.56 ± 13.05	43.45 ± 7.99	*T* = 1.76	0.087^a^
Gender (Male/Female)	11/7	10/10	χ^2^ = 0.47	0.532^b^
Education (years)	12.33 ± 2.85	13.75 ± 2.57	*T* = −1.61	0.116^a^

Data are represented as the mean ± SD. *p* < 0.05 was considered significant. pDoC, prolonged disorders of consciousness; HCs, healthy controls. ^a^p value obtained using an independent-sample *t*-test. ^b^*p* value obtained using a chi-square test.

### 3.2 White matter: intergroup differences in FA

**Figure 1:**
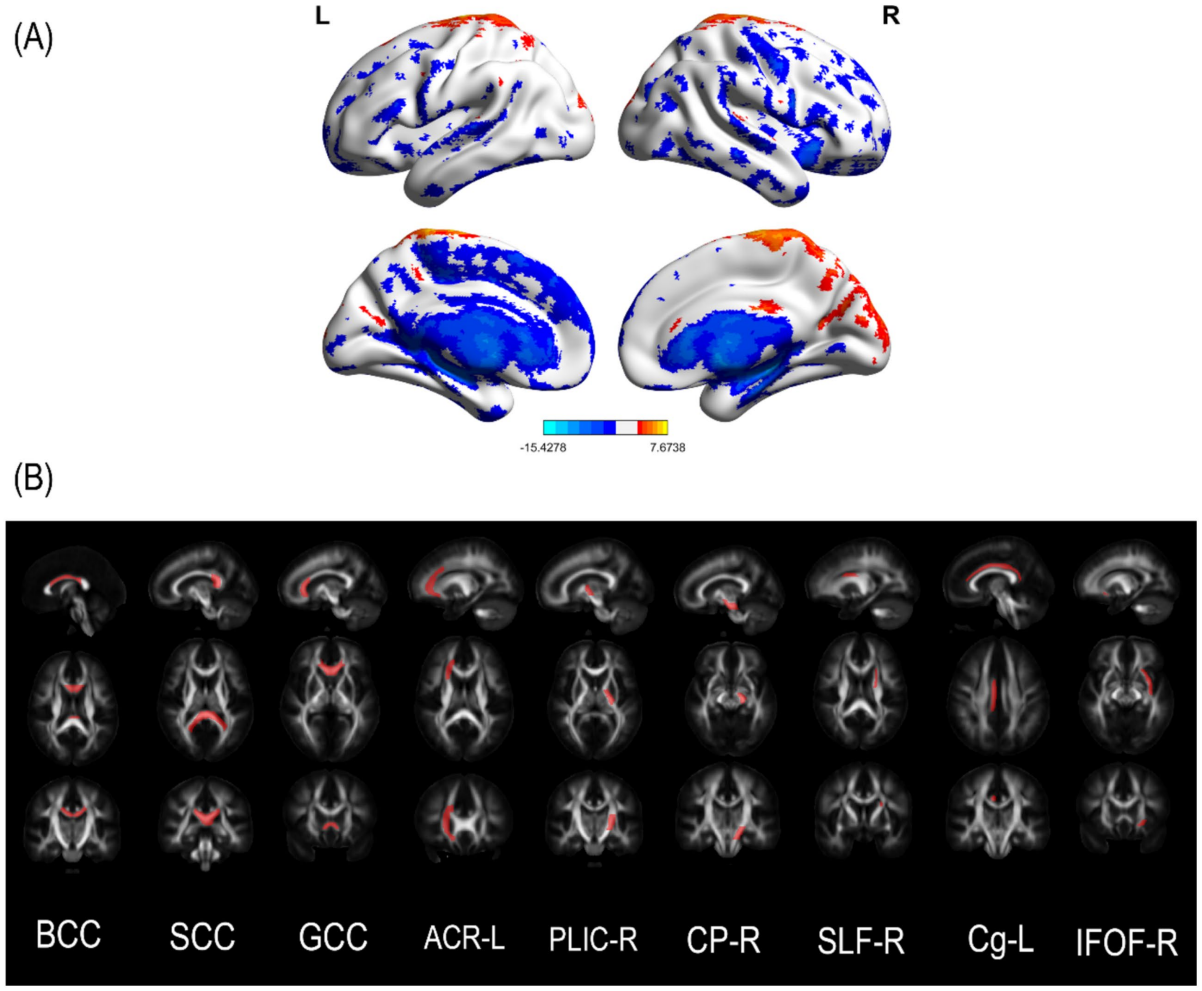
FA intergroup differences: White matter microstructure in pDoC patients vs. HCs. **(A)** Blue regions show brain areas with reduced FA in pDoC patients (TFCE-corrected *p* < 0.05). The peak coordinate of the cluster is at the left anterior corona radiata (ACR-L). **(B)** Quantitative comparison of FA values in nine key white matter tracts: boxplots compare FA values between pDoC patients (red) and HCs (green). Boxplots display the median, interquartile range, and distribution range. Significant intergroup differences were observed in the body of corpus callosum (BCC), splenium of corpus callosum (SCC), genu of corpus callosum (GCC), ACR-L, right posterior limb of internal capsule (PLIC-R), right cerebral peduncle (CP-R), right superior longitudinal fasciculus (SLF-R), left cingulum (Cg-L), and right inferior fronto-occipital fasciculus (IFOF-R). Asterisks indicate significance levels: **p ≤ 0.01, ***p ≤ 0.001.

Whole-brain voxel-wise analysis identified FA reductions across multiple white matter regions in pDoC patients relative to HCs. Statistical mapping identified a single large cluster (cluster voxel count = 107,385; peak MNI coordinates: [−22, 30, 4]; peak *t*-value = −15.428; TFCE-corrected *p* < 0.05), with peak coordinate at the ACR-L. The spatial extent of this cluster appears in Figure 1A, with detailed metrics provided in Table 3. This large cluster comprises nine key tracts (Figure 1B): corpus callosum body (BCC), splenium of corpus callosum (SCC), genu of corpus callosum (GCC), ACR-L, right posterior limb of internal capsule (PLIC-R), right cerebral peduncle (CP-R), right superior longitudinal fasciculus (SLF-R), left cingulum (Cg-L), and right inferior fronto-occipital fasciculus (IFOF-R). These tracts represent anatomical subregions within the widespread white matter cluster. No independent statistical test was performed to avoid circular analysis (double-dipping).

**Table 3:** Brain regions with significant intergroup differences in FA between pDoC patients and HCs.

Indices	Cluster	Number of voxels	Peak MNI coordinate	Peak MNI coordinate region	Label include (Voxels≥50)	Peak intensity (*t*-value)
FA						
	1	107,385	−22 30 4	ACR-L	BCC, SCC, GCC, SCR_R, ACR_L, ACR_R, SCR_L, SLF_R, SLF_L, PLIC_L, PLIC_R, ALIC_R, ALIC_L, CP_R, CP_L, Cg_L, Cg_R, IFOF_R, IFOF_L, Fx/ST_L, Fx/ST_R	−15.428

FA, fractional anisotropy; BCC, Body of corpus callosum; SCC, Splenium of corpus callosum; GCC, Genu of corpus callosum; ACR, Anterior corona radiata; SCR, Superior corona radiata; ALIC, Anterior limb of internal capsule; PLIC, Posterior limb of internal capsule; CP, Cerebral peduncles; SLF, Superior longitudinal fasciculus; Cg, Cingulum; Fx/ST, Fornix / Stria Terminalis; IFOF, Inferior Fronto-Occipital Fasciculus; L, Left; R, Right.

### 3.3 Intergroup differences in ALFF

**Figure 2:**
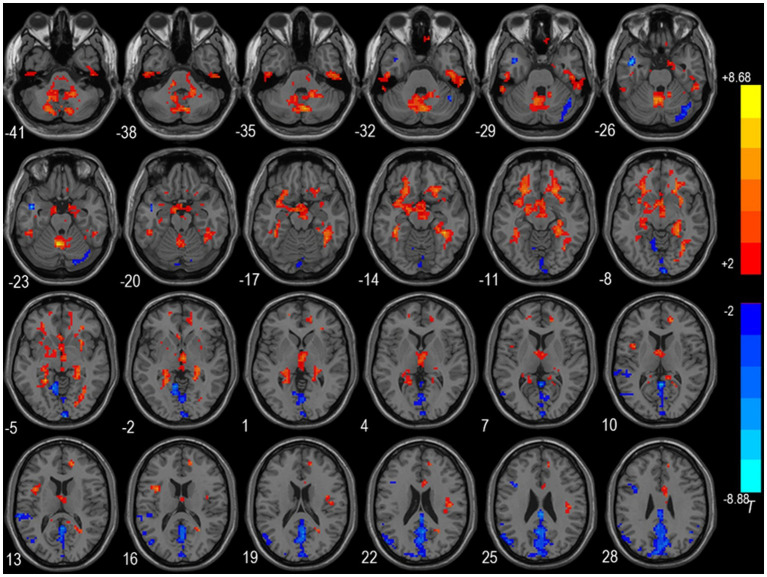
ALFF regional changes: pDoC patients vs. HCs. Brain regions with increased ALFF include bilateral cerebellar lobules VIII and IX (Cb8/9-L/R), and a right temporal cluster (FFG-R, ITG-R, Hipp-R). Regions with significantly reduced ALFF include the bilateral precuneus (PCun), cuneus (Cun), right angular gyrus (Angular-R), left inferior parietal lobule (IPL-L), and left angular gyrus (Angular-L) (TFCE-corrected *p* < 0.05). The color bar represents *t*-statistic values (TFCE corrected *p* < 0.05).

Group-level differences in ALFF were detected in the pDoC group relative to HCs (Table 4, Figure 2; TFCE-corrected *p* < 0.05). Compared with HCs, the pDoC group showed elevated ALFF in bilateral cerebellar lobules VIII and IX (Cb8/9) and a right temporal cluster including the right fusiform gyrus (FFG-R), right inferior temporal gyrus (ITG-R), and right hippocampus (Hipp-R). In contrast, ALFF was reduced in the bilateral precuneus (PCun), cuneus (Cun), right angular gyrus (Angular-R), left inferior parietal lobule (IPL-L), and left angular gyrus (Angular-L).

**Table 4:** Brain regions with significant intergroup differences in ALFF, fALFF, and ReHo between pDoC patients and HCs.

Indices	Cluster	Number of voxels	Peak MNI coordinate	Peak MNI coordinate region	Label include (Voxels≥50)	Peak intensity (*t*- value)
ALFF	1	1,477	−3 −81 33	Cun-L	PCun-L, Cun-L, PCun-R, Cun-R, Calcarine-L	−8.88
	2	953	−6 −57 −24	None	Cb8-L, Cb9-L, Cb9-R, Cb8-R	8.683
	3	530	36 –39 −15	FFG-R	FFG-R, ITG-R, Hipp-R	7.07
	4	166	45 –60 33 5	Angular-R	Angular-R	−5.93
	5	121	−42 −51 45	IPL-L	IPL-L	−6.77
	6	102	−48 −72 24	Angular-L	Angular-L	−5.62
fALFF	1	2,195	−15 −30 18	None	FFG-R, ITG-R, Hipp-R, Cb8-R, rPUT, CbCrus2-R	6.52
	2	454	3 51 21	SFGmed-R	SFGmed-R/L, SMA-L	−5.99
	3	221	−51 −60 −48	CbCrus2-L	CbCrus2-L	5.21
	4	205	−42 3 36	PreC-L	PreC-L, IFGtri-L	−5.80
ReHo	1	2,989	24 18 54	FSup2-R	FSup2-R, SFGmed-L, FMid2-R, FSup2-L, PreC-R, FInfTri-R, FMedOrb-L, ACC-sup-L, Ins-R, FInfOper-R, ACC-pre-L, FMid2-L, PostC-R	−6.453
	2	1,205	9 3 –33	None	PHG-L, Hipp-L, TPOmid-L, ITG-L	5.802
	3	428	45 −21 −33	FFG-R	ITG-R, FFG-R	7.256
	4	102	−39 −45 −18	ITG-L	ITG-L	5.6839

ALFF, Amplitude of Low-Frequency Fluctuation; fALFF, fractional Amplitude of Low-Frequency Fluctuation; ReHo, Regional Homogeneity; Cb, Cerebellar Lobule; Ver, Vermis; PCun, Precuneus; Cun, Cuneus; Calcarine, Calcarine Cortex; FFG, Fusiform Gyrus; PHG, Parahippocampal Gyrus; ITG, Inferior Temporal Gyrus; Hipp, Hippocampus; rPUT, Putamen_R; SMA-L, Supp_Motor_Area_L; IFGtri-L, Frontal-Inf-Tri-L; IPL, Inferior Parietal Lobule; SFGmed, Medial Superior Frontal Gyrus; TPOmid, Middle Temporal Pole; FSup2, Superior Frontal Gyrus 2; FMid2, Middle Frontal Gyrus 2; PreC, Precentral Gyrus; FInfTri, Triangular Part of Inferior Frontal Gyrus; FMedOrb, Medial Orbital Frontal Gyrus; ACC-sup, Superior Anterior Cingulate Cortex; Ins, Insula; FInfOper, Opercular Part of Inferior Frontal Gyrus; ACC-pre, Pregenual Anterior Cingulate Cortex; PostC, Postcentral Gyrus; L, Left; R, Right.

### 3.4 Intergroup differences in fALFF

**Figure 3:**
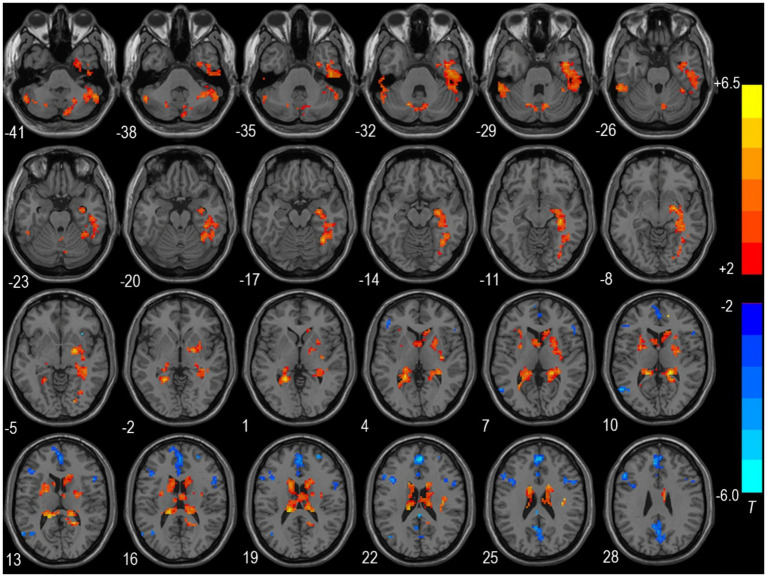
fALFF regional changes: pDoC patients vs. HCs. Brain regions with significantly increased fALFF include the right fusiform gyrus (FFG-R) and right hippocampus (Hipp-R). The region with significantly reduced fALFF involves the left medial superior frontal gyrus (SFGmed-L). The color bar represents *t*-statistic values, (TFCE-corrected *p* < 0.05).

fALFF exhibited between-group discrepancies in the pDoC group compared to HCs (Table 4, Figure 3; TFCE-corrected *p* < 0.05). Relative to HCs, the pDoC group showed elevated fALFF in a large cluster predominantly involving the right fusiform gyrus, right inferior temporal gyrus, right hippocampus, right cerebellar lobule VIII, right putamen, and right cerebellar crus II (peak MNI: −15, −30, 18; *t* = 6.52), as well as in the left cerebellar crus II (peak: −51, −60, −48; *t* = 5.21). Reduced fALFF was detected in the bilateral medial superior frontal gyrus and left supplementary motor area (peak: 3, 51, 21; *t* = −5.99), and in the left precentral gyrus and left inferior frontal gyrus, triangular part (peak: −42, 3, 36; *t* = −5.80).

### 3.5 Intergroup differences in ReHo

**Figure 4:**
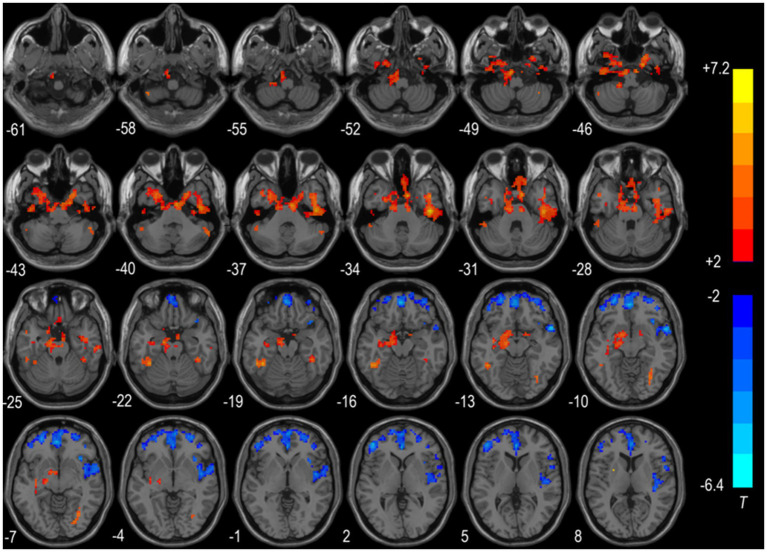
ReHo regional changes: pDoC patients vs. HCs. Brain regions with significantly increased ReHo include the left parahippocampal gyrus (PHG-L), left hippocampus (Hipp-L), right fusiform gyrus (FFG-R), and left inferior temporal gyrus (ITG-L). Regions with significantly reduced ReHo involve an extensive prefrontal cluster, including the right superior frontal gyrus 2 (FSup2-R), SFGmed-L, bilateral middle frontal gyrus 2 (FMid2-L/R), bilateral inferior frontal gyrus, triangular part (FInfTri-L/R), bilateral medial orbital frontal gyrus (FMedOrb-L/R), right precentral gyrus (PreC-R), right postcentral gyrus (PostC-R), left superior anterior cingulate cortex (ACC_sup-L), right insula (Ins-R), and right middle temporal pole (TPOmid-R). The color bar represents *t*-statistic values, (TFCE-corrected *p* < 0.05).

ReHo displayed distinct between-group variations in pDoC patients compared to HCs (Table 4, Figure 4; TFCE-corrected *p* < 0.05). Regions with reduced ReHo formed an extensive prefrontal cluster, encompassing the right superior frontal gyrus 2 (FSup2-R), SFGmed-L, bilateral middle frontal gyrus 2 (FMid2-L/R), bilateral inferior frontal gyrus, triangular part (FInfTri-L/R), bilateral medial orbital frontal gyrus (FMedOrb-L/R), right precentral gyrus (PreC-R), right postcentral gyrus (PostC-R), left superior anterior cingulate cortex (ACC-sup-L), right insula (Ins-R), and right middle temporal pole (TPOmid-R). Regions with elevated ReHo included three clusters: one in the left medial temporal lobe covering the left PHG (PHG-L) and left Hipp (Hipp-L); one in the right FFG (FFG-R); and one in the left inferior temporal gyrus (ITG-L).

### 3.6 Intergroup differences in FC

**Figure 5:**
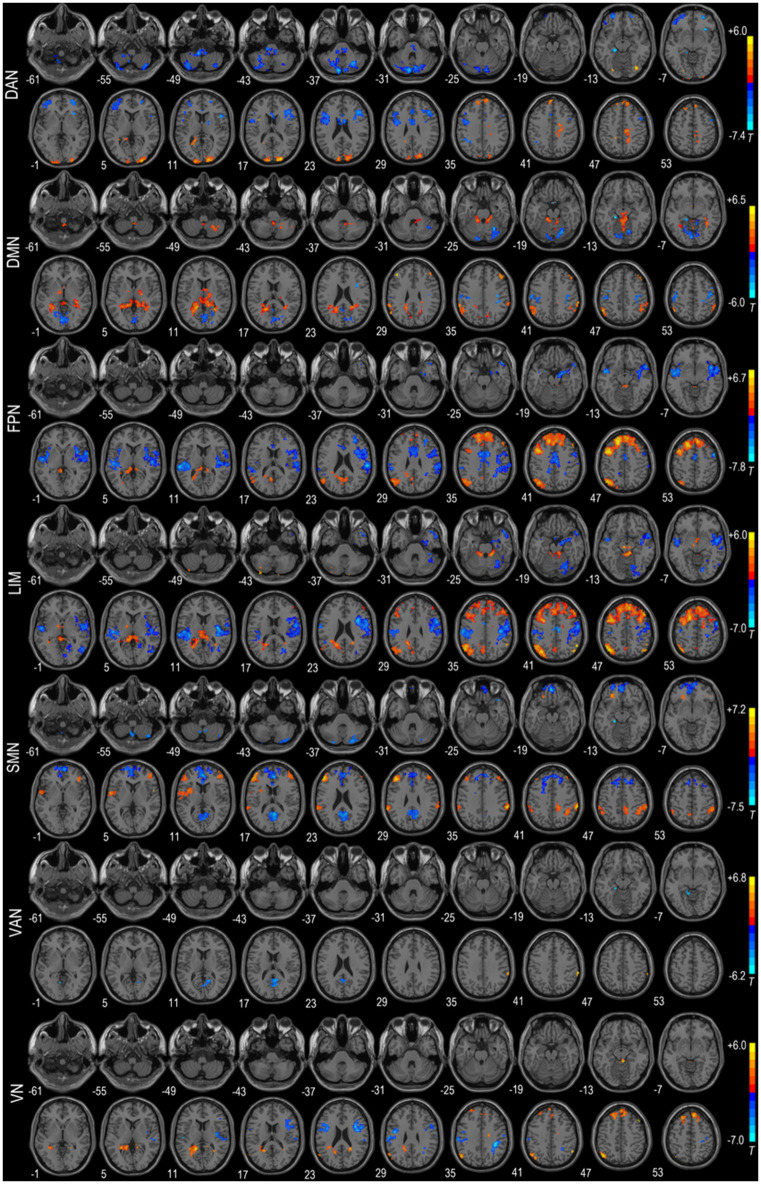
Intergroup differences in functional connectivity (FC): PDoC patients vs. HCs. Intra-network FC was significantly reduced across multiple higher-order networks, with enhanced connections between the right cerebellar lobule III (Cb3-R) and left angular gyrus (Angular-L) in the default mode network (DMN), limbic network (LIM), and frontoparietal network (FPN). FC was decreased in the right supramarginal gyrus (SMG-R), left superior temporal gyrus (TSup-L), and right middle cingulate gyrus (CMid-R) but increased in the left superior frontal gyrus 2 (FSup2-L) and Angular-L within the FPN. Within the dorsal attention network (DAN), FC reductions were observed in the FSup2-L, left precentral gyrus (PreC-L), and left cerebellar Crus 2 (CbCrus2-L), with increased connectivity in the right superior occipital gyrus (Occipital-Sup-R). Diminished FC was identified in the left postcentral gyrus (PostC-L), right PreC-R, and right cerebellar lobule VI (Cb6-R) alongside increased connectivity in the Cb3-R and left inferior parietal lobule (IPL-L) within the DMN. In the LIM, FC was reduced in the right insula (Ins-R), left TSup-L, and left PostC-L but augmented in the FSup2-L, Cb3-R, and Angular-L. Within the sensorimotor network (SMN), FC declines were noted in the left rectus gyrus (Rectus-L), right precuneus (PCun-R), and right CbCrus2-R, with enhanced connectivity in the bilateral middle frontal gyrus 2 (FMid2-L/R), SMG-R, and left rolandic operculum (RolOper-L). The ventral attention network (VAN) showed reduced FC in the right calcarine cortex (Calcarine-R). In the visual network (VN), FC was diminished in the PreC-L and elevated in the right medial superior frontal gyrus (SFGmed-R) and Angular-L. The color bar represents *t*-statistic values, (TFCE-corrected *p* < 0.05).

Resting-state FC analysis of seven classic networks revealed significant alterations across various networks in patients with pDoC compared to HCs (TFCE-corrected *p* < 0.05; Figure 5; Table 5). Patients exhibited significantly diminished intra-network FC across various higher-order networks. While both the right cerebellar lobule III (Cb3-R) and left angular gyrus (Angular-L) demonstrated enhanced connectivity within the DMN, LIM, and FPN.

**Table 5:** Intergroup differences in functional connectivity (FC): cluster characteristics and peak statistics in pDoC patients vs. HCs.

ROI	Cluster	Number of voxels	Peak MNI coordinate	Peak MNI coordinate region	Peak intensity (*t*- value)
FPN	1	1,967	60 –27 24	SMG-R	−7.156
	2	1,610	–18 33 51	FSup2-L	6.705
	3	696	–60 –30 12	TSup-L	−7.770
	4	605	9 –21 39	CMid-R	−5.670
	5	413	–45 –66 48	Angular-L	6.009
DAN	1	1,085	–9 –84 –36	CbCrus2-L	−7.234
	2	455	15 –96 21	Occipital-Sup-R	5.990
	3	328	–42 –3 33	PreC-L	−5.707
	4	298	–24 54 –9	FSup2-L	−5.644
DMN	1	1,372	9–36 -15	Cb3-R	5.573
	2	774	27 –60 –24	Cb6-R	−5.360
	3	328	–27 –30 69	PostC-L	−5.141
	4	204	–54 –57 48	IPL-L	5.699
	5	162	45 –15 57	PreC-R	−5.113
	6	138	66 –36 42	SMG-R	5.410
LIM	1	2,320	36 –9 12	Ins-R	−6.590
	2	2,188	–18 18 51	FSup2-L	5.661
	3	732	9 –36 –15	Cb3-R	5.865
	4	492	51 –21 9	TSup-L	−6.980
	5	474	–39 –72 42	Angular-L	5.994
	6	439	12 –78 –6	Lingual-R	−4.723
	7	316	–57 –18 39	PostC-L	−5.663
SMN	1	1,589	–3 51 –18	Rectus-L	−7.452
	2	436	6 –57 18	PCun-R	−6.192
	3	301	66 –36 36	SMG-R	7.189
	4	247	–42 36 30	FMid2-L	6.930
	5	214	–57 0 3	RolOper-L	5.823
	6	200	48 45 9	FMid2-R	4.930
	7	124	27 –81 –36	CbCrus2-R	−5.726
VAN	1	125	15 –54 12	Calcarine-R	−4.967
VN	1	333	9 42 51	SFGmed-R	5.927
	2	156	–45 –6 33	PreC-L	−5.625
	3	115	–45 –66 48	Angular-L	5.874

FPN, Frontoparietal Network; DAN, Dorsal Attention Network; DMN, Default Mode Network; LIM, Limbic Network; SMN, Sensorimotor Network; VAN, Ventral Attention Network; VN, Visual Network; CbCrus2, Cerebellar Crus 2; Cb, Cerebellar Lobule; Occipital-Sup, Superior Occipital Gyrus; PreC, Precentral Gyrus; FSup2, Superior Frontal Gyrus 2; PostC, Postcentral Gyrus; IPL, Inferior Parietal Lobule; SMG, Supramarginal Gyrus; TSup, Superior Temporal Gyrus; CMid, Middle Cingulate Gyrus; Angular, Angular Gyrus; Ins, Insula; Lingual, Lingual Gyrus; Rectus, Rectus Gyrus; PCun, Precuneus; FMid2, Middle Frontal Gyrus 2; RolOper, Rolandic Operculum; Calcarine, Calcarine Cortex; SFGmed, Medial Superior Frontal Gyrus; L, Left; R, Right.

In the FPN, FC was diminished in the right supramarginal gyrus (SMG-R), left superior temporal gyrus (TSup-L), and right middle cingulate gyrus (CMid-R), whereas connectivity was augmented in the left superior frontal gyrus 2 (FSup2-L) and Angular-L. In the DAN, FC decreased in the FSup2-L, left precentral gyrus (PreC-L), and left cerebellar Crus 2 (CbCrus2-L), while it increased in the right superior occipital gyrus (Occipital-Sup-R). Reduced FC was observed in the left postcentral gyrus (PostC-L), right precentral gyrus (PreC-R), and right cerebellar lobule VI (Cb6-R) within the DMN, accompanied by enhanced connectivity in the Cb3-R and left inferior parietal lobule (IPL-L). Within the LIM, reduced FC was found in the right insula (Ins-R), left TSup-L, and left PostC-L, whereas the FSup2-L, Cb3-R, and Angular-L exhibited augmented connectivity. In the SMN, FC decreased in the left rectus gyrus (Rectus-L), right precuneus (PCun-R), and CbCrus2-R, while it increased in the bilateral middle frontal gyrus 2 (FMid2-L/R), SMG-R, and left rolandic operculum (RolOper-L). The VAN exhibited diminished FC in the right calcarine cortex (Calcarine-R). In the VN, FC decreased in the PreC-L and increased in the right medial superior frontal gyrus (SFGmed-R) and Angular-L.

### 3.7 Correlations with CRS-R scores

**Figure 6:**
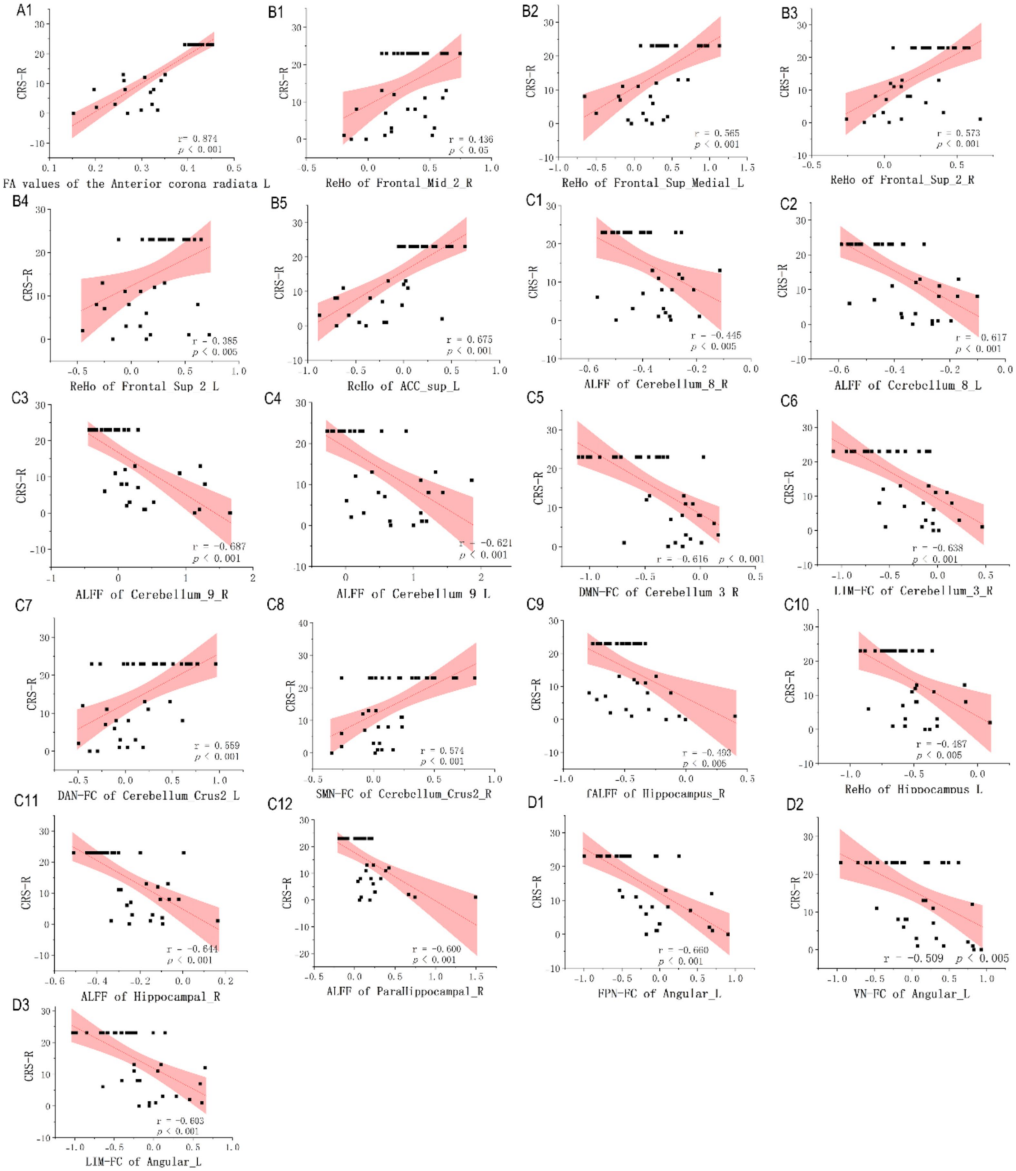
Correlation between neuroimaging measures and CRS-R scores in patients with pDoC. **(A1)** FA in the left anterior corona radiata (ACR-L) was positively correlated with CRS-R scores (*r* = 0.874, *p* < 0.001). **(B1–B5)** ReHo in prefrontal regions was positively correlated with CRS-R scores: right middle frontal gyrus 2 (FMid2-R) (*r* = 0.436), left medial superior frontal gyrus (SFGmed-L) (*r* = 0.565), right superior frontal gyrus 2 (FSup2-R) (*r* = 0.573), left superior frontal gyrus 2 (FSup2-L) (*r* = 0.385), and left superior anterior cingulate cortex (ACC-sup-L) (*r* = 0.675). **(C1–C4)** ALFF in cerebellar regions was negatively correlated with CRS-R scores: right/left cerebellar lobule VIII (Cb8-R/L) (*r* = −0.445/−0.617) and right/left cerebellar lobule IX (Cb9-R/L) (*r* = −0.687/−0.621). **(C5,C6)** FC of the right cerebellar lobule III (Cb3-R) in the DMN/LIM was negatively correlated with CRS-R scores (*r* = −0.616/−0.638). **(C7,C8)** FC of the left cerebellar Crus 2 (CbCrus2-L) in the DAN and right cerebellar Crus 2 (CbCrus2-R) in the SMN was positively correlated with CRS-R scores (*r* = 0.559/0.574). **(C9–C12)** Right hippocampus (Hipp-R) ALFF (*r* = −0.644) and fALFF (*r* = −0.493), left hippocampus (Hipp-L) ReHo (*r* = −0.487), and right parahippocampal gyrus (PHG-R) ALFF (*r* = −0.600) were negatively correlated with CRS-R scores. **(D1–D3)** FC of the left angular gyrus (Angular-L) in the FPN/VN/LIM was negatively correlated with CRS-R scores (*r* = −0.660/−0.509/−0.603). (All *p* < 0.05).

Partial correlation adjusted for UWS/MCS subtype showed a strong positive association between ACR-L FA and CRS-R scores (*r* = 0.874, *p* < 0.001). ReHo values in several prefrontal subregions also correlated positively with CRS-R scores (*p* < 0.05): the right FMid2 (*r* = 0.436), SFGmed-L (*r* = 0.565), right FSup2 (*r* = 0.573), left FSup2 (*r* = 0.385), and left ACC_sup (*r* = 0.675). Several indices from the cerebellum and limbic system, however, showed negative correlations with CRS-R scores. ALFF in bilateral Cb8 (right: *r* = −0.445; left: *r* = −0.617) and Cb9 (right: *r* = −0.687; left: *r* = −0.621), as well as in the Hipp-R (*r* = −0.644) and PHG-R (*r* = −0.600), were negatively associated with CRS-R scores (*p* < 0.05). FC of the Cb3-R within the DMN (*r* = −0.616) and LIM (*r* = −0.638), and FC of the Angular-L within the FPN (*r* = −0.660), VN (*r* = −0.509), and LIM (*r* = −0.603), all exhibited robust negative correlations with CRS-R scores (*p* < 0.005). FC of the CbCrus2-L within the DAN (*r* = 0.559) and CbCrus2-R within the SMN (*r* = 0.574) were positively correlated with CRS-R scores (*p* < 0.001) (see Figure 6).

## 4 Discussion

This multimodal investigation uncovers a coherent set of structural and functional brain abnormalities in pDoC. Findings show different types of changes that include reduced white matter properties, altered patterns of activity in specific regions, and reduced connections between large networks in the brain. These changes are not a series of events that happen one after the other; they are all happening at the same time. They include widespread damage to white matter structure, less neural activity and network integration in higher-order cortical areas, and hyperconnectivity in subcortical areas. This study shows a three-tiered concurrent pathological framework that gives us a new way to think about the pathophysiology of pDoC.

### 4.1 Structural disconnection

We found significant FA decreases in many white matter areas in pDoC patients, with these changes widespread across deep white matter with peak coordinate at ACR-L. This widespread microstructural damage involves key long-range tracts including the corpus callosum, internal capsule, superior longitudinal fasciculus and cerebral peduncles, highlighting the critical role of white matter integrity in sustaining consciousness (35). Our findings align with prior evidence that thalamocortical projection integrity predicts recovery potential after severe brain injury (36). In fact, microstructural damage to thalamocortical and intercortical pathways is the core pathophysiological mechanism underlying consciousness impairment (37). The body, splenium, and genu of the corpus callosum exhibit significant reductions in FA, indicating impaired interhemispheric connectivity and functional coordination. This observation offers structural neuroimaging evidence for the hypothesis that consciousness relies on synchronized activity across extensive brain networks, as the integration of information between hemispheres is crucial for the formation of a cohesive conscious experience. Beyond this, damage to descending projection fibers, such as those within the posterior limb of the internal capsule and the cerebral peduncle, may further weaken functional linkages between the cortex and subcortical structures, including the brainstem and spinal cord, this degradation likely increases the disintegration of the main network that processes information. These observations are consistent with prior reports emphasizing a positive correlation between the integrity of thalamocortical pathways and the level of consciousness (38). As the central hub for information exchange between the thalamus and prefrontal cortex, the ACR-L coordinates the transmission of thalamic sensory inputs, modulates cortical arousal states, and facilitates the conveyance of higher-order cognitive signals. Problems in this pathway disrupt the movement of signals in the area but also affect the main system that maintains the state of being awake. Multiple studies show relationships between the degree of consciousness recovery in comatose patients and the reconstruction of ascending arousal network connectivity (39), our findings further indicate that global diffuse white matter disruption with peak coordinate at ACR-L impairs both arousal signaling and awareness-related content. Partial correlation adjusted for UWS/MCS subtype demonstrated a robust positive correlation between ACR-L FA values and CRS-R scores, underscoring the region’s significance in consciousness-related networks and providing a clearer insight into the effects of pathway damage on clinical outcomes.

### 4.2 Higher-order cortical networks disintegration

Beyond white matter structural disruption, we observed significant reductions in local neural activity and synchrony in pDoC patients specifically in cortical regions critical for self-referential processing and environmental perception. This functional impairment is marked by widespread decreases in ALFF, fALFF and ReHo across the bilateral precuneus, cuneus and extensive prefrontal cortices. The precuneus is a core default mode network node that supports self-awareness and memory integration. Reduced neural activity here directly impairs patients’ ability to self-monitor and engage with their surroundings, and aligns with established theory linking default mode network integrity to subjective selfhood (40, 41), while providing an objective neuroimaging correlate for pDoC’s defining feature: lack of consistent, purposeful interaction with self or environment.

As a key neural substrate, the prefrontal cortex supports goal-directed actions and underlies various higher-order cognitive processes. Patients with pDoC exhibited widespread ReHo decreases, implying a lack of local neural synchronization that impairs coordination of higher cognitive processes. Of note, the SFGmed-L is a critical frontoparietal network node involved in executive function and exhibited selective fALFF decreases, the impaired neural activity in this region may directly contribute to patients’ deficits in following commands and engaging in goal-directed interactions (42). Previous research supports the link between intact prefrontal function and behavioral responsiveness in consciousness disorders and provides an objective basis for distinguishing different awareness states. At the network level, FC analysis revealed widespread reductions in core node connections within the FPN, DAN and DMN consistently with consciousness research consensus. Reduced FPN connectivity involving the SMG-R and TSup-L points to executive and integrative function deficits. Impaired DMN connectivity affecting the FSup2-L and PreC-L undermines attentional allocation. Concurrent DMN dysfunction exacerbates self-related cognitive processing and episodic remembering impairments. The extensive static FC disruption reflects severe impairment in the brain’s ability to coordinate across systems. Conscious awareness depends on a dynamic balance between global integration and functional diversity (43). The collapse of both within- and between-network connectivity within the FPN and DMN provides a direct illustration of this mechanism breakdown and offers an explanation for lost conscious content in pDoC. Correlation analyses revealed positive correlations between ReHo values in multiple subregions of prefrontal cortex and CRS-R scores, indicating that local neural synchrony in higher-order cortices is critically important for the presence and level of consciousness. Our results extend previous reports linking prefrontal function to impairment severity and add nuance from subregional analysis to understanding how different prefrontal areas may contribute to consciousness, highlighting that functional integrity in these regions is clinically important.

### 4.3 Excessive connectivity of the cerebellum and limbic system and the three concurrent pathological patterns

In stark contrast to the hypoactivity observed in higher-order cortical networks, patients with pDoC exhibited elevated neuroimaging metrics within the cerebellum and limbic system. Specifically, we found considerably increased ALFF in Cb8/9. Concurrently, ALFF in the right hippocampus and parahippocampal gyrus, fALFF in the right hippocampus, putamen, and cerebellum (Cb8, Crus2), and ReHo in the left hippocampus and parahippocampal gyrus all displayed upward trends. Furthermore, abnormally enhanced FC was observed involving the Angular-L and Cb3-R. These patterns of regional hyperactivity and hyperconnectivity may be driven by several mechanisms: reduced top-down inhibitory control from the prefrontal cortex, transneuronal diaschisis secondary to widespread cortical disconnection, and global network hyperexcitability. Alternatively, they may reflect a form of targeted but maladaptive functional compensation. First, the heightened activity observed in Cb8/9 appears to align with its proposed role in cognitive-affective processing via cerebello-thalamo-cortical circuits (44, 45). Additionally, aberrant network connectivity of Cb3-R might correspond to its function as a node in cortical networks (46), which seems consistent with prior evidence that the cerebellar anterior lobe may regulate arousal transitions through Purkinje cell-mediated thalamocortical pathways (45). The observed hyperactivity and hyperconnectivity show significant negative correlations with CRS-R scores, reflecting the severity of network dysregulation (16).

Regarding hippocampal alterations, it fulfills a vital dynamic function across the complete trajectory of disorders of consciousness. Given that acute-phase studies have established preserved hippocampal function as a positive biomarker for recovery (44, 47), the significant hyperactivity observed in the pDoC hippocampus and associated cortices in this study, which negatively correlated with clinical scores. These findings are consistent with stage-dependent functional reorganization rather than conflicting with previous reports. Against the backdrop of persistent disintegration of higher-order neural networks in the chronic phase, the hippocampus may transition from a “functional support node” to a “dysregulated compensatory state.” Thus, chronic localized hyperactivity likely reflects abnormal activation triggered by disrupted network homeostasis. This activity worsens overall functional disorder instead of helping with conscious integration.

The left angular gyrus showed unusually high FC across several networks, a finding negatively correlated with CRS-R scores. Our findings appear different from those of Laureys et al., which may be due to differences in disease stage, patient chronicity, and analytic methods, Laureys et al. (48) said that people with DOC had less connectivity in the angular gyrus, while Liudmila et al. (49) said that stimulating the angular gyrus could improve consciousness. This discrepancy likely stems from critical stage-dependent and network-state-dependent factors. Intervention studies typically involve subacute patients whose underlying structural connectivity may be partially preserved. In contrast, the chronic-phase patients in our study showed widespread white matter disconnection. In this context, chronic deafferentation may drive a shift from functional inhibition to maladaptive hyperconnectivity: heightened local activity here signals pathological dysfunction, rather than efficient cross-regional information integration.

Drawing on the findings of this study on white matter structure, higher cortical networks, and subcortical compensatory systems, the pathogenesis of pDoC may present as three concurrent pathological patterns: widespread white matter disruption, cortical network disintegration, and subcortical maladaptive changes. This maladaptive stage not only fails to restore the dynamic global integration necessary for consciousness, but further disrupts brain network balance. In the chronic phase, there is local neuronal hyperactivity and abnormally strengthened connectivity. These coexisting patterns are associated with sustained consciousness impairment (50).

### 4.4 Ascending arousal system dysfunction in pDoC

Consciousness depends on the brainstem, hypothalamus, thalamus, and their connecting white matter pathways. Together these structures form the ascending arousal system (AAS). Damage to the AAS is a well recognised cause of disorders of consciousness. Snider et al. used diffusion MRI to track AAS pathways and found that comatose patients had about 20% fewer connections from the brainstem tegmentum to the thalamus, hypothalamus and basal forebrain (51). Edlow et al. (52) mapped these pathways in detail in post mortem human brains. In chronic disorders of consciousness, Li et al. (16) confirmed that AAS structural connectivity is clearly disrupted and can separate patients from healthy controls. Our findings are consistent with these reports. We observed widespread FA reduction in white matter, with the left anterior corona radiata (ACR L) as a key tract. Lower FA in the ACR L was strongly linked to lower CRS R scores (*r* = 0.874). Because the ACR L connects the thalamus to the prefrontal cortex, its damage likely weakens the arousal signal travelling from the brainstem to the cortex.

The brainstem tegmentum is the origin of the AAS. Parvizi and Damasio showed that damage to specific tegmental nuclei in the midbrain can directly cause coma (52). Spindler et al. found that dopaminergic disconnection from the ventral tegmental area to the forebrain via the medial forebrain bundle (MFB) occurs in both reversible (anaesthesia) and chronic (DoC) unconsciousness (53). Coenen et al. (54) described the detailed anatomy of the MFB using diffusion tensor imaging. Our study did not directly reconstruct the MFB or perform detailed segmentation of tegmental nuclei. However, the broad FA reduction we observed in the ACR L and surrounding white matter – areas close to the MFB – indirectly suggests that these pathways may also be affected. Dedicated tractography and higher resolution imaging are needed to confirm this.

Numerous studies have shown that functional connectivity between the thalamus and the cortex is crucial for consciousness (4, 40, 55). Cosgrove ME et al. founded that (36) thalamo-prefrontal connectivity in the return of goal-directed behavior following TBI (36). Li et al. (16) observed widespread loss of functional connectivity between several thalamic nuclei and the cortex in patients with DoC (55). Using 7T fMRI, Edlow et al. showed that subcortical hubs in the brainstem and thalamus are functionally connected to both the default mode and salience networks (56). Our FC results showed reduced connectivity within higher order networks (default mode, frontoparietal, limbic, etc.) and abnormally enhanced connectivity of the right cerebellar lobule III and left angular gyrus. These patterns are compatible with the idea that insufficient ascending input from the thalamus leads to cortical network disintegration. The hyperconnectivity of the angular gyrus and cerebellum might be a maladaptive response to chronic deafferentation.

Taken together, previous literature and our multimodal findings (structural FA, local activity ALFF/fALFF/ReHo, and functional connectivity) consistently point to a bottom-up dysfunction of the ascending arousal system in pDoC. While our results are compatible with the current model, they also suggest the potential coexistence of multiple levels of abnormality, from white matter disconnection to disintegration of cortical networks and regional hyperactivity in chronic patients. The overall pattern supports the idea that reduced arousal input to the cortex contributes to the impairment of consciousness.

### 4.5 Strengths and limitations

A notable aspect of this work lies in the conjoint utilization of multimodal neuroimaging metrics and behavioral assessments. We identified three concurrent tiers of pathological co-alterations in pDoC. These concurrent patterns link structural white matter alterations to functional network breakdown and maladaptive excessive connectivity within subcortical regions. It offers objective biomarkers to evaluate pDoC injury severity, recovery potential, and maladaptive compensatory mechanisms. Extensive research has demonstrated that neuromodulation of the medial thalamus and prefrontal cortex enhances behavioral reactivity (57, 58), Our results align with these prior observations. They confirm the thalamus and prefrontal cortex’s critical role in consciousness recovery. Further inspired mechanism-based dual-target neuromodulation strategies. The approach involves stimulating key nodes in the prefrontal cortex or default mode network. It aims to restore network function. Meanwhile, it suppresses hyperactive hubs like the angular gyrus or cerebellar lobules VIII/IX. This action disrupts the pathological balance. ACR-L integrity consistently correlates with clinical CRS-R scores, which supports these findings. It highlights this specific structural hub’s value as a prognostic tool.

Despite these contributions, this study has notable limitations. MRI acquisition requires patients to stay motionless. Unsedated pDoC patients often exhibit involuntary movements. Examples include oral chewing and head motion. Even among patients with stable vital signs, occasional drops in oxygen saturation may occur. These happen during transport from the ward to the imaging suite. They lead to high data rejection rates. This in turn restricts the cohort size. The cross-sectional study design and etiological heterogeneity of the patient population hinder our capacity. We cannot establish causal or temporal links among the coexisting pathological patterns. This may obscure pathological features specific to different subtypes. While multimodal MRI effectively characterizes macroscopic structural and functional changes, it still provides insufficient insight into neurotransmitter dynamics and neuroinflammatory responses. In addition, standard MNI template spatial normalization may introduce subtle anatomical bias in patients with chronic cerebral atrophy and ventricular enlargement. Although nonlinear registration and atrophy masking were applied, potential distortion-related effects on group-level analyses remain an inherent limitation of the present study. Our focus on pDoC leaves a key question unanswered. Similar mechanisms may or may not apply to other consciousness-related disorders. This limits the generalizability of our results. To address these limitations, future research will collaborate across multiple centers. The goal is to expand the sample size and boost statistical power. We will adopt a longitudinal study design. It will track changes of structure–function-compensation patterns over time. We will stratify analyses by etiology and disease stage. This helps uncover potential subtype-specific pathological patterns. And we will use PET or MRS combined with multimodal MRI to explore the neurochemical basis of compensation. And the targeted intervention trial based on the dual-target strategy will be key. Longitudinal follow-up cohort studies and interventional trials are required for further clinical translation of these imaging biomarkers. So as to turn our theoretical framework into clinical application.

## 5 Conclusion

DTI measurements of FA values reveal widespread reductions in FA in white matter tracts, with peak coordinate at the ACR-L, in patients with pDoC. rs-fMRI studies demonstrate abnormalities in ALFF/fALFF/ReHo within the prefrontal cortex, cerebellum, and limbic regions, alongside disrupted FC within higher cortical networks. These findings demonstrate three concurrent pathological co-alterations in pDoC: widespread microstructural damage in the white matter, with peak coordinate at the ACR-L; the disintegration of higher-order cortical networks; patterns of hyperactivity and hyperconnectivity in the cerebellum, limbic system, and angular gyrus. The neuroimaging biomarkers found in this study provide a comprehensive framework for analyzing the pathophysiological mechanism of consciousness disorders, and also provide new ideas for the objective evaluation, prognosis judgment and precise targeted therapy of pDoC.

## Data Availability

The original contributions presented in the study are included in the article/supplementary material, further inquiries can be directed to the corresponding author.
